# Respiratory involvement in ambulant and non-ambulant patients with facioscapulohumeral muscular dystrophy

**DOI:** 10.1007/s00415-017-8525-9

**Published:** 2017-05-26

**Authors:** Sandra Moreira, Libby Wood, Debbie Smith, Chiara Marini-Bettolo, Michela Guglieri, Grace McMacken, Geraldine Bailey, Anna Mayhew, Robert Muni-Lofra, Gail Eglon, Maggie Williams, Volker Straub, Hanns Lochmüller, Teresinha Evangelista

**Affiliations:** 1 Serviço de Neurologia, Centro Hospitalar Entre Douro e Vouga, Santa Maria da Feira, Portugal; 2 John Walton Muscular Dystrophy Research Centre, MRC Centre for Neuromuscular Diseases, Institute of Genetic Medicine, Newcastle University, Central Parkway, Newcastle upon Tyne, NE1 3BZ UK; 3 Bristol Genetics Laboratory, Southmead Hospital, North Bristol NHS Trust, Bristol, UK

**Keywords:** Facioscapulohumeral dystrophy, Respiratory impairment, Restrictive lung function

## Abstract

Understand the occurrence and predictors of respiratory impairment in FSHD. Data from 100 FSHD patients was collected regarding demographics, genetics, respiratory status and pulmonary function tests, clinical manifestations and Clinical Severity Scale (CSS) scores. Patients were assigned to two severity groups using CSS: mild (scores <3.5) and moderate/severely affected (scores ≥3.5). Forced Vital Capacity (FVC) was classified as severely impaired if less than 50% of the predicted. Statistical analysis was performed using IBM SPSS Statistics 23, tests were two-tailed and the level of significance set at 5%. Spirometry was available for 94 patients; 41.5% had abnormal results with a restrictive pattern in 38.3% patients. There was a correlation between FVC; CSS score and D4Z4 fragment length with a higher probability of severe respiratory involvement in the early onset group, moderate/severe disease and D4Z4 fragments <18 kb. Patients with severe respiratory involvement showed a high prevalence of sleep-disordered breathing. FVC decline over time was indicative of three progression groups. Respiratory involvement for both ambulant and non-ambulant patients with FSHD is more frequent and severe than previously suggested. Sleep-disordered breathing is frequent and negatively influences the respiratory status. Annual screening of the respiratory status with spirometry and clinical assessment is thus warranted in FSHD patients, even while ambulant.

**Electronic supplementary material:**

The online version of this article (doi:10.1007/s00415-017-8525-9) contains supplementary material, which is available to authorized users.

## Introduction

Facioscapulohumeral dystrophy (FHSD) is the third most common muscular dystrophy, with overall prevalence of 3.2–4.6/100,000 [1, 2]. Despite its typical onset with facial and shoulder weakness, FSHD shows striking clinical heterogeneity [3–5]. Early onset tends to be associated with shorter D4Z4 fragments, faster progression and more severe phenotype [3, 6, 7].

FSHD results from different mechanisms occurring in carriers of a permissive 4qA allele that lead to hypomethylation of the 3.3 kb D4Z4 repeat array in chromosome 4q35, chromatin relaxation and pathologic expression of DUX4 [8]. D4Z4 repeat contraction causes disease in 95% of cases (FSHD1); in 5% (FSHD2) the mechanism is for most related to mutations in *SMCHD1* [3, 8, 9].

Shorter D4Z4 repeat arrays are associated to higher penetrance and earlier disease onset [7, 10, 11]. Some studies hinted a negative correlation between the fragment size and severity of disease [10, 12, 13]; while the association between short arrays and high severity scores is consistent, longer repeats show a higher variability [3, 10, 11, 14, 15].

Respiratory involvement (RI) is acknowledged as a rare manifestation of FSHD [17–22] and is described as mild and occurring late in the course of the disease, with only 1–3% of the patients requiring non-invasive ventilation (NIV) [17, 22]. The risk of RI seems higher in patients with spine deformities, severe disease, weaker proximal lower limbs and wheelchair dependency [17, 19–23]. Sleep-disordered breathing (SDB), including hypoventilation syndrome (HS) and obstructive sleep apnoeas (OSAS), are frequent in FSHD patients, may occur early in the course of the disease and may have a subclinical presentation. There is apparently no correlation between the presence of SDB and the disease severity [22, 24, 25].

The aim of this study is to evaluate the frequency and severity of RI in FSHD, identify predisposing factors for respiratory failure and reasons for NIV, and characterize the progression of forced vital capacity (FVC) before and after NIV, and produce evidence to raise standards of care in FSHD.

## Materials and methods

### Patients

The current retrospective study included 100 genetically confirmed FSHD patients followed at the John Walton Muscular Dystrophy Research Centre (JWMDRC), for whom data was collected regarding: age of onset, age of last assessment, muscle strength, locomotor independency severity scores according to the CSS [14], spine deformities, dysphagia, respiratory symptoms, symptoms and type of SDB and spirometry results over time.

Patients were assigned to two severity groups, according to the CSS score: mildly affected group scores <3.5 and moderate/severely affected group scores ≥3.5. Males and females were compared in terms of severity scores and FVC levels. The 10-grade CSS scale was used taking into account the extent of weakness in various muscular regions, and considering the spread of symptoms from face to upper limb, pelvic and leg muscles. According to this scale [14], higher scores were ascribed to patients with a more pronounced involvement of pelvic and proximal lower limb muscles, for this we used the muscle assessments reported in the patients’ notes.

Genetic confirmation was performed using Southern blot-based methods for FSHD1 [9] and hypomethylation screening plus SMCHD1 sequence analysis for FSHD2 [8].

Patients were divided in two groups for age of onset (data available for 88 patients) using Brouwer’s criteria [26]: early onset and normal onset. Both groups were compared for severity scores, frequency of wheelchair dependency, D4Z4 fragment size and respiratory function. Duration of the disease was calculated based on age of last assessment and age of onset.

We have also compared patients with D4Z4 fragments sizes above or below 18 kb (corresponding to 4-unit fragments) [13], as this cut-off allowed for statistical significance in differences between the two groups.

### Respiratory function

Respiratory function was assessed through clinical data and spirometry results (collectively available for 94 patients). Spirometry was performed using Microlab Care Fusion portal spirometer with facial mask. Patients with FVC <80% predicted, normal FEV1/FVC ratio (≥70%) and no symptoms or clear risk of obstructive lung disease were classified as having a restrictive pattern. Two patients with a mixed pattern were included in this group given the predominant restrictive component. An obstructive pattern was considered in patients with FEV1 <80% and FEV1/FVC ratio <70% [27, 28]. For further detail on respiratory function analysis, see supplementary material.

Patients with sustained FVC <50% predicted or with FVC at the last assessment <50% not explained by an acute intercurrence, were classified as severe respiratory involvement group (SR group) and compared to the non-severe respiratory involvement group with FVC ≥50% (NSR group).

Diagnosis of NH (with desaturation or hypercapnia) was based on overnight pulse oximetry results and arterial gases. The diagnosis of OSAS was done by polysomnography. Symptoms of SDB were considered when patients complained of excessive daytime sleepiness, snoring, apnoeas, morning headaches or morning sickness.

### Statistical analysis

Demographics and population characteristics were reported using descriptive statistics. The Chi-square test was performed to assess differences between groups. Independent samples *t* test and Kruskal–Wallis test were used to compare means between groups. A logistic regression model was used and odds ratio calculated to compare the risk of wheelchair dependency in two different groups of age of onset.

The correlation between variables (CSS, D4Z4 repeat size, FVC levels, age of onset and disease duration) was done using simple linear regression and Pearson’s correlation coefficient (Pearson’s *r*), provided that residuals followed normal distribution. Stepwise multiple regression analysis was used to build a predictive model of FVC levels. To further assess the risk of having a restrictive respiratory pattern and severe RI in different groups, logistic regression models were built and odds ratios estimated.

Simple linear regression was performed to characterize FVC decline in SR group, based on the slope of the regression line of FVC values. Ten out of 14 patients were considered suitable for this characterization, as they had at least two FVC measures 3 years apart. Mean of the individual slopes was assumed as the mean FVC decline. To compare FVC progression before and after NIV start, fourteen patients under NIV (8-SR group; 6-NSR group) were further analysed through visual analysis of individual plots of FVC levels.

Statistical tests were two-tailed and the level of significance was set at 5%. Statistical analysis was performed using IBM SPSS Statistics 23 for Windows.

## Results

### Clinical and genetic results

We collected data on 100 genetically confirmed FSHD patients from 61 families, 54 males and 46 females. Ninety-seven patients had FSHD type 1 and 3 had FSHD type 2; in 81/97 we were able to obtain the size of the D4Z4 fragment, while in the remaining 16 we only had information on a confirmed pathological range of the fragment size. The mean age of disease onset (*n* = 88 patients) was 23.8 ± 15.7 years (23.2 ± 15.3 for FSHD1 and 39.0 ± 21.8 for FSHD 2, *p* = 0.336). Six patients were classified as early onset and 82 as normal onset. The mean age at the last assessment was 48 ± 16.7 years with mean disease duration of 22.8 ± 15.0 years.

Using CSS, 46 patients were classified as mildly affected and 54 as moderate/severely affected, 14 of which were wheelchair bound (supplementary material: Figure S1.a). There was a positive correlation between the duration of symptoms and the CSS (*r* = 0.603, *p* < 0.001) (Supplementary material: Figure S1.b). Males and females did not differ in terms of severity scores (*p* = 0.273). Distribution of severity scores in the early onset and normal onset groups was significantly different (*p* = 0.021). The early onset group included a patient with isolated facial weakness, one who walked with assistance (score 4.5) and four wheelchair-bound patients, which is a significantly higher proportion than that observed in the normal onset group (66.7 vs 20.7%, *p* = 0.028). This translates into a 7.7 times higher probability of becoming wheelchair dependent in the early onset group (odds ratio 95% IC 1.3–45.3, *p* = 0.025).

Spine deformities, mostly hyperlordosis (*n* = 13), were present in 18% of patients; two patients had scoliosis and kyphosis; one had scoliosis and one kyphosis. The majority of patients with spine deformities (55.6%) were wheelchair bound, compared to 19.5% of patients without spine deformities.

**Fig. 1:**
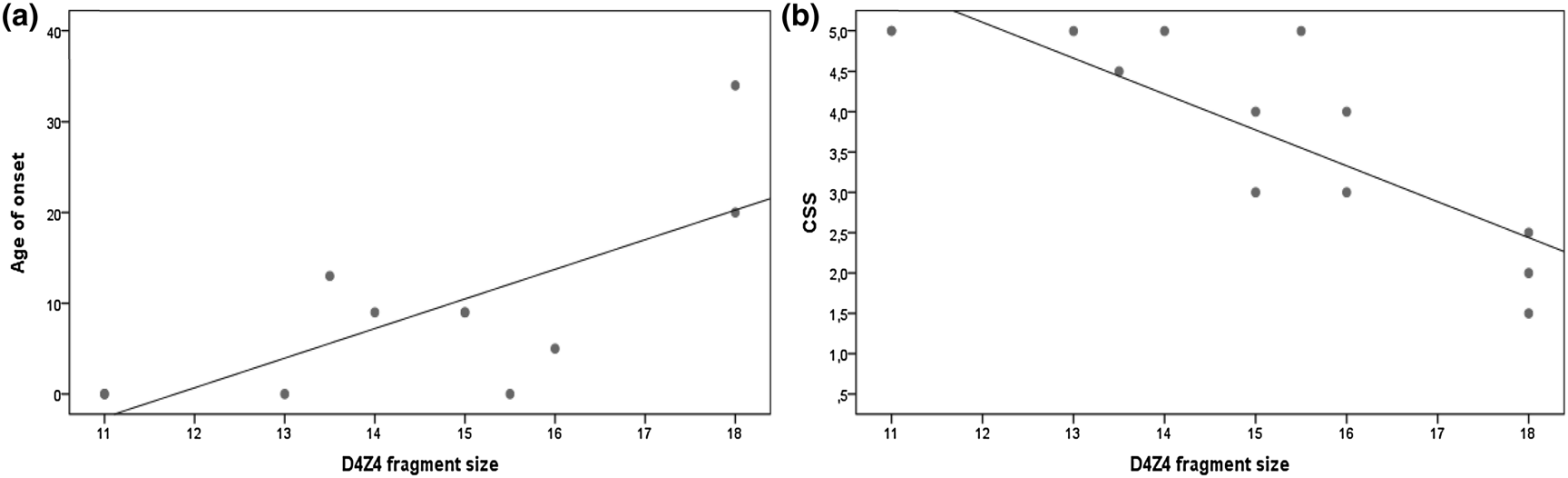
Correlation between D4Z4 fragment size and **a** age of onset (*r* = 0.737, *p* = 0.01) and **b** Clinical Severity Scale scores (*r* = −0.838, *p* < 0.01), in patients with fragments up to 18 kb

When considering the patients with D4Z4 fragments <18 kb, a significant linear correlation was found between the fragment size and both age of onset (*r* = 0.737, *p* = 0.01) (Fig. 1a) and severity scores (*r* = −0.838, *p* < 0.01) (Fig. 1b). Patients with fragments in this range showed a higher proportion of early onset when compared to patients with D4Z4 fragments >18 kb (66.7 vs 33.3%, *p* = 0.005). There was no difference in the number of moderate/severe cases (61.5 vs 58.8%, *p* = 0.855) in these two groups.

### Respiratory assessment

Spirometry results were available in 94 patients. There was no difference in FVC between genders (*p* = 0.397); 55 patients (58.5%) showed normal spirometry results, 36 (38.3%) had a restrictive pattern and 3 (3.2%) an obstructive pattern. In 14 patients, FVC levels were <50% (SR group), corresponding to 14.9% of all patients and to 38.9% of the restrictive pattern group.

**Fig. 2:**
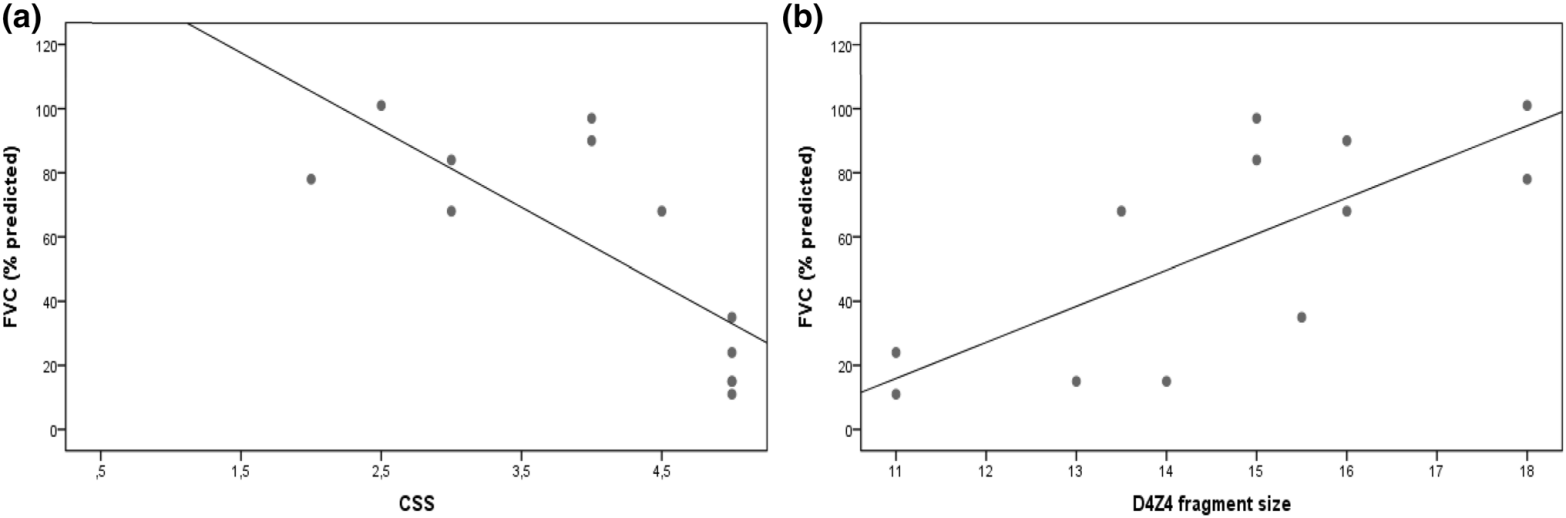
**a** Correlation between CSS scores and FVC (*r* = −0.770, *p* = 0.03). **b** Correlation between D4Z4 fragment size and FVC (*r* = 0.745, *p* = 0.005), in patients with fragments up to 18 kb

A linear correlation was found between the CSS and the FVC values (*r* = −0.770, *p* = 0.03) for patients with D4Z4 fragments <18 kb (Fig. 2a). Mean FVC was significantly lower in patients with moderate/severe disease than with mild disease (mean FVC 69.0 ± 24.5 vs 92.2 ± 15.4%, *p* < 0.001), the former had a significantly higher probability of restrictive lung disease (odds ratio 5.5, 95% IC 2.1–14.5, *p* = 0.001) and severe RI (odds ratio 12.4, 95% IC 1.5–99.0, *p* = 0.018). The D4Z4 size showed a linear correlation with the FVC levels (*r* = 0.745, *p* = 0.005) for fragments <18 kb (Fig. 2b). Compared to patients with larger fragments, those with fragments <18 kb had a 4.9× higher probability of having severe RI (odds ratio 95% IC 1.3–19.3, *p* = 0.022).

An earlier age of onset showed tendency towards worse respiratory outcome than the normal onset group (table S2), as shown by the lower mean FVC values (40.2 ± 31.4 vs 80.0 ± 22.6%, *p* = 0.026), the higher frequency of restrictive pattern (83.3 vs 32.9%, *p* = 0.023) and severe respiratory compromise (66.7 vs 11.8%, *p* = 0.005). Logistic regression showed a 10.2× higher probability of developing a restrictive pattern (odds ratio 95% IC 1.1–92.0, *p* = 0.039) and a 14.9× higher probability of having a drop in FVC below 50% (odds ratio 95% IC 2.4–93.2, *p* = 0.04) in the early onset compared to the normal onset group.

A predictive model for FVC decline was built by multiple regression analysis considering 62 patients with known age of onset, spirometry results and D4Z4 repeat size. A significant linear correlation was found between FVC, severity scores (*r* *=* −0.607, *p* < 0.001) and D4Z4 fragment size (*r* = 0.422, *p* = 0.004) when adjusting for age of onset (*r* = 0.205, *p* = 0.618). The disease duration did not improve the predictive model or altered the correlation coefficient of the other variables. Multiple regression analysis considering only patients with D4Z4 fragments <18 kb showed that the correlation between fragment size and FVC loses significance when adjusting for severity, which in turn maintains a significant correlation with FVC (*r* = −0.846, *p* = 0.002).

Differences between SR and NSR groups are depicted in Table 1. Compared to NSR, the SR group showed lower mean D4Z4 fragment sizes (19.8 ± 6.5 vs 25.7 ± 6.1 kb, *p* = 0.008) and a higher proportion of moderate/severe cases (92.9 vs 51.2%, *p* = 0.04), higher percentage of wheelchair-bound patients (71.4 vs 20.0%, *p* < 0.001) and of patients with spine deformities (50.0 vs 13.8%, *p* = 0.005), but not of dysphagia (17.5 vs 21.4%, *p* = 0.729). Table 2 summarizes respiratory function and associated comorbidities in patients belonging to the SR group and in patients with SDB belonging to the NSR group. Seventeen patients were diagnosed with SDB, including 13 with NH, two with OSAS and two with NH and OSAS. One patient with NH with hypercapnia and another with OSAS were asymptomatic. Compared to patients without SDB, patients with SDB showed lower FVC values (45.2 ± 4.1 vs 83.5 ± 2.3%, *p* < 0.001), higher frequency of severe RI (58.8 vs 5.2%, *p* < 0.001) and of moderate/severe cases (100 vs 44.6%, *p* < 0.001).

**Table 1:** Characteristics of NSR group and SR group

	NSR group	SR group	*p*
Respiratory pattern, *n* = 94			
Normal, *n* (%)	55 (68.8%)	0	
Obstructive, *n* (%)	3 (3.7%)	0	
Restrictive, *n* (%)	22 (27.5%)	14 (100%)	
NIV, *n* (%)	6	8	
D4Z4 repeat array, *n* = 75			
Mean (kb)	25.7 ± 6.1	19.8 ± 6.5	0.008
Range (kb)	14–40	11–30	
Odds ratio for SR^a^		4.9 [1.3–19.3]	0.022
11–18 kb, *n* (%)	7 (11.3%)	5 (38.5%)	0.029
19–40 kb, *n* (%)	55 (88.7%)	8 (61.5%)	
CSS, *n* = 94			
Mild, *n* (%)	39 (48.8%)	1 (7.1%)	0.004
Moderate to severe, *n* (%)	41 (51.2%)	13 (92.9%)	
Locomotor independency, *n* = 94			
Ambulant, *n* (%)	64 (80.0%)	4 (28.6%)	<0.001
Wheelchair dependent, *n* (%)	16 (20.0%)	10 (71.4%)	
Spine deformities, *n* = 94			
Yes, *n* (%)	11 (13.8%)	7 (50.0%)	0.005
No, *n* (%)	69 (86.3%)	7 (50.0%)	
Dysphagia, *n* = 94			
Yes, *n* (%)	14 (17.5%)	3 (21.4%)	0.713
No, *n* (%)	66 (82.5%)	11 (78.6%)	

NSR group (non-severe respiratory involvement) and SR group (severe respiratory involvement) percentages in brackets refer to the proportion within each group

*NIV* non-invasive ventilation, *SCC* severity clinical score

^a^Odds ratio refers to probability of severe respiratory involvement in patients with D4Z4 fragments up to 18 kb compared to patients with fragments larger than 18 kb

**Table 2:** Respiratory function and associated complications of all patients in the SR group and of patients with SDB in the NSR group

Patient number (#)	Age of onset (years)	Duration of disease	Time of FVC follow-up (years)	FVC (% predicted)		FVC decline (% per year)	SDB			Symptoms		Respiratory infections*	Smoker	NIV	Duration of disease until NIV
				Max	Min		NH Desaturations	NH hypercapnia	OSAS	SDB	Dyspnoea				
SR group															
1	<1	23	6	40	11	4.8	–	Yes	–	–	Yes	–	–	BiPAP trach	14
13	57	5	2	43	40	–	Yes	–	Yes	Yes	–	Yes	–	BiPAP N	6
23	9	31	1	20	15	–	Yes	–	–	Yes	Yes	Yes	–	BiPAP trach	26
27	46	30	11	55	33	1.6	–	Yes	–	Yes	Yes	Yes	Yes	CPAP N/D	28
29	40	11	1	56	43	–	–	Yes	–	Yes	Yes	Yes	Yes	CPAP noct	10
31	5	47	16	75	42	1.5	Yes	–	–	Yes	Yes	Yes	Yes	CPAP N/D	34
81	11	43	1	38	32	–	–	Yes	Yes	Yes	–	Yes	–	CPAP N	42
100	12	39	3	36	22	4.7	Yes		–	Yes	–	–	Yes	BiPAP N	38
68	<1	58	9	53	35	1.9	–		–	–	–	–	–	–	–
74	<1	33	9	31	15	1.5	–		–	–	–	–	–	–	–
76	–	–	3	54	38	4.8	Yes		–	Yes	Yes				–
84	<1	23	12	55	24	2.1	–		–	–	–	–	–	–	–
86	37	12	8	62	49	1.2	–		–	–	–	–	–	–	–
87	6	54	3	76	38	12.1	Yes		–	Yes	Yes	–	–	–	–
NSR group															
41	20	45	1	53	–	–	Yes	–	–	Yes	–	–	–	CPAP N	44
43	4	43	6	73	51	2.8	–	–	Yes	–	–	–	–	CPAP N	40
47	25	25	14	90	70	0.9	Yes	–	–	Yes	–	–	–	CPAP N	24
54	25	32	10	60	50	0.8	–	–	Yes	Yes	Yes	Yes	Ex-smoker	CPAP N	32
61	–	–	11	92	38	3.5	Yes	–	–	Yes	–	–	–	CPAP N	–
80	22	41	7	83	60	2.5	Yes	–	–	Yes	–	–	–	CPAP N	34
52	34	12	2	92	71	–	Yes	–	–	Yes	–	–	Yes	–	–

FVC values correspond to maximum and minimum registered during the respiratory follow-up period. For patient #61 duration of the disease until NIV is not displayed because of unknown age of onset (age at NIV start was 54 years). “Respiratory infections” refer to pneumonia-needing antibiotics, recurrent chest infections or chest infection leading to respiratory failure. (*NH* nocturnal hypoventilation, *SDB* sleep-disordered breathing, *NIV* non-invasive ventilation)

**Fig. 3:**
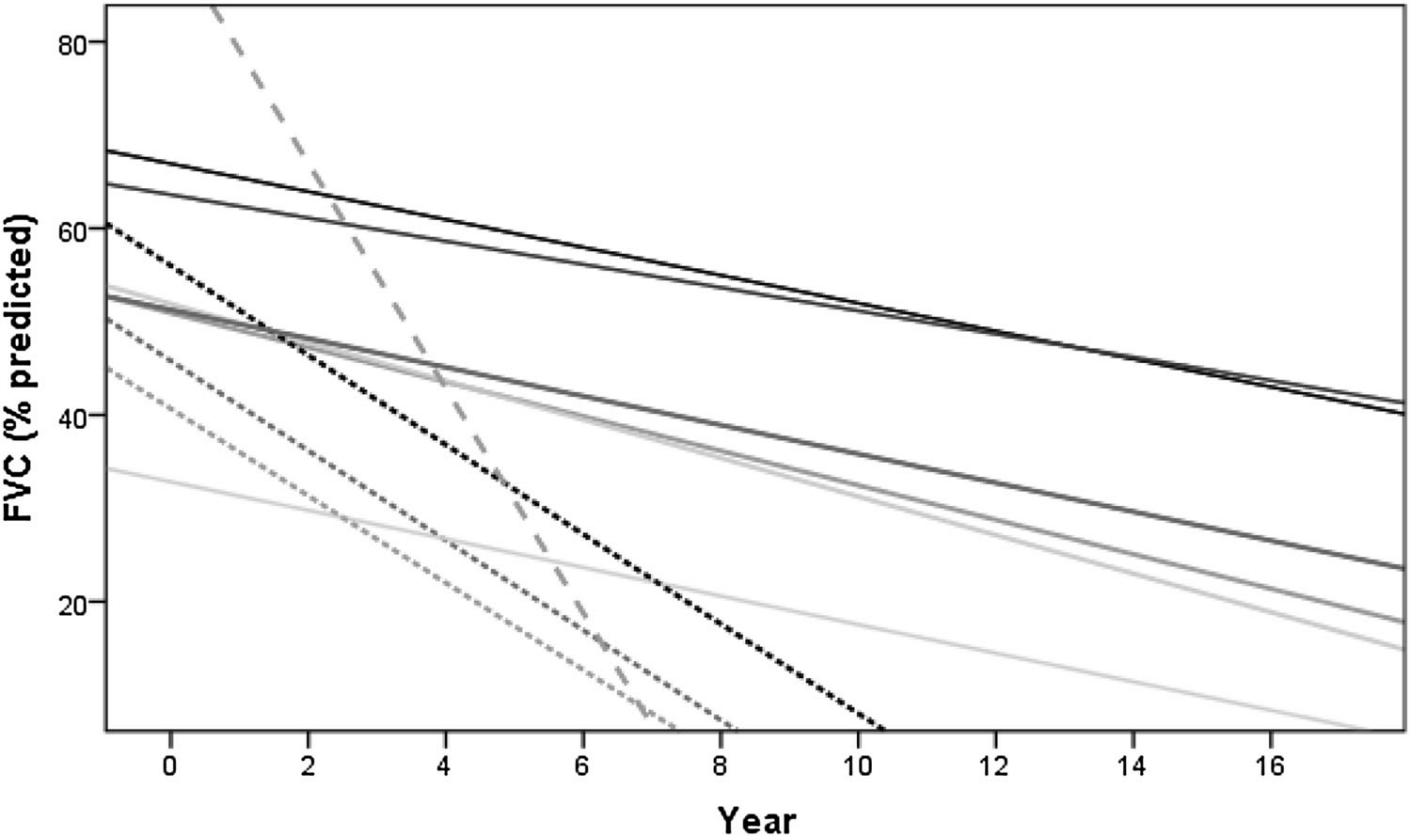
FVC decline over time in patients who developed severe respiratory involvement (*n* = 10). *Lines* correspond to individual patient’s FVC regression lines. *Full lines* represent patients with a slower decline (type 1 progression, mean decline 1.6 ± 0.3%/year), *spotted lines* represent patients with a faster decline (type 2 progression, mean decline 4.8 ± 0.1%/year) and *dashed line* represents a single patient with an extremely fast decline (decline 12.1%/year)

The FVC values over time in ten patients from the SR group showed a decline that ranged between 1.2 and 12.1% per year, with a mean of 3.6 ± 3.3% (Fig. 3). The duration of respiratory follow-up, maximum and minimum recorded FVC values and percentage of FVC decline per year in each patient are shown in Table 2. Visual analysis of all the ten regression lines and their slopes (Fig. 3) suggested three types of significantly different progression rates (*p* = 0.032): a slower FVC decline between 1.2 and 2.1% per year (mean 1.6 ± 0.3%) (Type 1 progression); a faster decline between 4.7 and 4.8% per year (mean 4.8 ± 0.1%) (Type 2 progression); and a single patient who showed an even faster decline of 12.1% per year over a 4-year period (Type 3 progression).

Fourteen patients were started on NIV, eight of whom belonged to the SR group and six to the NSR. FVC values in patients under NIV (figures S2.1 to S.15) showed a trend towards stabilization or even improvement and a trend towards a new deterioration if NIV was stopped. Mean age and mean duration of disease at NIV were 50.07 ± 14.5 and 28.6 ± 2.3 years, respectively. An FVC drop to 22% predicted prompted to start BiPAP in one patient previously diagnosed with NH that did not tolerate CPAP. In the remaining patients of the SR group, NIV was started after acute respiratory failure in 3, after the diagnosis of NH in 3 and after diagnosis of NH and OSAS in one. Two patients refused NIV despite symptomatic NH. CPAP was initiated in five patients with SDB belonging to the NSR group; four had NH and one asymptomatic OSAS. One patient recently diagnosed with NH with desaturations was due to start NIV.

## Discussion

This study includes the largest FSHD population with respiratory assessment reported so far. The proportion of FSHD2 patients is slightly lower than previously reported [3, 5, 8, 9]. The positive correlation between disease duration and severity scores confirms the progressive nature of the disease. In agreement with other studies [3, 7], we confirmed a higher proportion of wheelchair users in the early onset group, with a prevalence that is remarkably high (66.7%) comparing to what was previously reported (4.2–21%) [7]. Despite the small number of patients with early onset, the prevalence may even be underestimated, as 18 cases with undetermined age of onset (5 non-ambulant) were excluded from the analysis.

Our results support previous suggestions that shorter D4Z4 arrays are associated with an earlier onset [11], as shown by the higher proportion of early onset cases in patients with D4Z4 fragments <18 kb. However, the linear correlation between D4Z4 size and age of onset was only present when considering fragments <18 kb, with larger fragments this is more unpredictable.

Overall, patients with shorter fragments did not have significantly more severe disease than patients with larger ones, which may reflect the clinical variability and the high proportion of severe cases that was observed in the latter group. In the former, we found a significant linear correlation between fragment size and disease severity, which agrees with previous studies showing that correlation with severity, is more consistent for shorter repeats, while subjects carrying longer repeats have a high variability in clinical severity [3, 10, 11, 14–16]. According to the literature, carriers of D4Z4 fragments >6 units may have disproportionate low methylation levels depending not only on the fragment size but also on epigenetic mechanisms, such as co-inheritance of SMCHD1 mutations [11]. We may speculate that unidentified disease modifiers may influence the age of onset and severity of disease, contributing to the clinical variability, especially in patients carrying larger arrays.

Our results show that RI is more frequent and severe than previously suggested [17, 20, 21]. Around 40% of our patients had a reduced respiratory function with a predominantly restrictive pattern. The prevalence of restrictive pulmonary involvement (38.3%) was higher than previously reported [2, 20] and unlike other studies [17, 19, 20] the respiratory compromise was severe and the use of NIV (14% of cases) was more frequent [18, 22].

In patients with D4Z4 fragments up to 18 kb, the strong correlation between fragment size and severity scores (Fig. 1b) and between severity scores and FVC (Fig. 2a) suggests that the association of smaller fragments with lower FVC levels may be explained, at least in part, by its influence on severity.

Patients in the SR group carried D4Z4 fragments smaller than patients in the NSR group. The wide range and overlap of fragment sizes in between groups supports the weak, yet significant association between size of the deletion and respiratory function. As shown by the higher probability of severe RI in patients with fragments <18 kb (odds ratio 4.9; 95% IC 1.3–19.3, *p* = 0.022).

Although severity of muscle weakness showed the strongest correlation with FVC values it is not very informative in early stages. Early disease onset and D4Z4 fragment size may be more useful as they allow prompt identification of patients at-risk of RI.

SDB was frequent in our population but the prevalence may have been underestimated due to the frequent subclinical presentation [24, 25] and insufficient screening of patients at-risk. Despite the significantly lower mean FVC in patients with SDB, approximately half of the patients with NH with desaturations and of those with OSAS still had FVC >50%, meaning that SDB may occur in the absence of severe RI, even with FVC levels as high as 70%. Given that all patients with SDB had severity scores above 3, it would be reasonable to investigate for NH and OSAS, asymptomatic patients with sustained FVC decline and lower limbs weakness, in addition to symptomatic patients regardless.

Analysis of the FVC decline (Fig. 3) in the SR group suggested three progression rates: slow decline 1.2–2.1% per year (type 1); fast decline 4.7–4.8% per year (type 2); and one patient with faster decline of 12.1% per year over a 4-year period (type 3). The slower FVC decline in type 1 cannot be explained by the inclusion of FVC measures >55% and a hypothetical slower decline in earlier stages of the disease, as it also included patients with FVC as low as 31%. A faster decline in type 2 and 3 does not seem to be related to smoking habits, a higher proportion of early onset cases or lung disease, as these were more frequent in type 1 (Table 3). The most frequent reason to start NIV was SDB, followed by acute respiratory failure. NIV should be started early in the course of the disease, when patients first have nocturnal hypoventilation (symptomatic or asymptomatic). This is substantiated by the trend towards stabilization or increase in FVC values once NIV is started.

## Conclusion

The comprehensive evaluation of a single-centre FSHD cohort suggests that RI is more frequent and severe than previously suggested and the restrictive pattern is the most frequent. Although our data suggest three progression rates of FVC decline, the small number of patients included in this analysis requires further validation.

Asymptomatic patients are at risk of developing SDB and acute respiratory failure, and may benefit from a more proactive screening of respiratory compromise. Patients should be actively checked for SDB due to its frequency and the negative effect on respiratory function. Patients with small D4Z4 arrays, early onset and moderate/severe disease have a higher risk of RI. Although CSS scores have the strongest correlation with the risk of RI, array size and age of onset may be more useful in the clinical practice allowing an earlier identification of at-risk patients.

## Electronic supplementary material

Below is the link to the electronic supplementary material.

Supplementary material 1 (DOCX 295 kb)
