# Supplementary material for: Respiratory involvement in ambulant and non-ambulant patients with facioscapulohumeral muscular dystrophy
Source: J Neurol. 2017 May 26;264(6):1271–80. doi: 10.1007/s00415-017-8525-9 (PMC5486574; doi:10.1007/s00415-017-8525-9)

**Supplementary material**

**Respiratory function**: Although restrictive lung disease is characterized by a reduction in total lung capacity (TLC) below the 5th percentile of the predicted value with a normal forced expiratory volume in 1 s/vital capacity (FEV1/FVC) ratio ([Pellegrino *et al.*, 2005](file:///D:\Neurologia\John%20Walton%20MDC\FSHD\Respiratory%20involvement%20FSHD\Manuscript%20for%20publication\Respirtaory%20involvement%20FSHD%20LAST.docx#_ENREF_18)), both TLC and data for percentiles calculus were absent from this population’s registries and workup. As there are no validated recommendations for NIV use in muscular dystrophies, the authors extrapolated from 2010 NICE guidelines for motor neuron disease management ([2010](file:///D:\Neurologia\John%20Walton%20MDC\FSHD\Respiratory%20involvement%20FSHD\Manuscript%20for%20publication\Respirtaory%20involvement%20FSHD%20LAST.docx#_ENREF_1)) in order to reasonably adapt 2005 ATS/European Respiratory Society (ERS) task force for interpretation of lung function tests ([Pellegrino *et al.*, 2005](file:///D:\Neurologia\John%20Walton%20MDC\FSHD\Respiratory%20involvement%20FSHD\Manuscript%20for%20publication\Respirtaory%20involvement%20FSHD%20LAST.docx#_ENREF_18)), which state that a reduced vital capacity may be suggestive of lung restriction when FEV1/VC is normal or increased. Allowing for NICE guidelines which consider patients with FVC less than 50% or FEV1 less than 80% predicted plus any symptoms or signs of respiratory impairment as candidates for NIV commencement, the authors found it reasonable and clinically useful to classify patients with FVC less than 80% predicted, normal FEV1/FVC ratio (higher or equal to 70%) and no symptoms or clear risk of obstructive lung disease as having a suggestive restrictive pattern (restrictive group). Two patients with a mixed pattern were included in this group given the predominant restrictive component. According to 2004 NICE guidelines for the diagnosis and management of chronic obstructive pulmonary disease (COPD)([Pearson, 2004](file:///D:\Neurologia\John%20Walton%20MDC\FSHD\Respiratory%20involvement%20FSHD\Manuscript%20for%20publication\Respirtaory%20involvement%20FSHD%20LAST.docx#_ENREF_17)), an obstructive pattern was considered in patients with FEV1 less than 80% and FEV1/FVC ratio less than 70% (obstructive group).

**Table S1** – Severity of facioscapulohumeral dystrophy according Clinical Severity Scale (CSS).

| 0.5 | Facial weakness. |
| --- | --- |
| 1 | Mild scapular involvement without limitation of arm abduction; no awareness of disease symptoms is possible. |
| 1.5 | Moderate involvement of scapular and arm muscles or both (arm abduction >60° and strength >3 in arm muscles); no involvement of pelvic and leg muscles. |
| 2 | Severe scapular involvement (arm abduction <60°on at least one side); strength <3 in at least one muscular district of the arms; no involvement of pelvic and leg muscles. |
| 2.5 | Tibioperoneal weakness; no weakness of pelvic and proximal leg muscles. |
| 3 | Mild weakness of pelvic and proximal leg muscles or both (strength >4 in all these muscles); able to stand up from a chair without support. |
| 3.5 | Moderate weakness of pelvic and proximal leg muscles or both (strength >3 in all these muscles); able to stand up from a chair with monolateral support. |
| 4 | Severe weakness of pelvic and proximal leg muscles or both (strength <3 in at least one of these muscles); able to stand up from a chair with double support; able to walk unaided. |
| 4.5 | Unable to stand up from a chair; walking limited to several steps with support; may use wheelchair for most activities. |
| 5 | Wheelchair bound. |

**Figure S1** – (a) Distribution of patients according to Clinical Severity Scale (CSS) scores and (b) correlation between duration of disease and CSS scores (r = 0.603, p < 0.001).


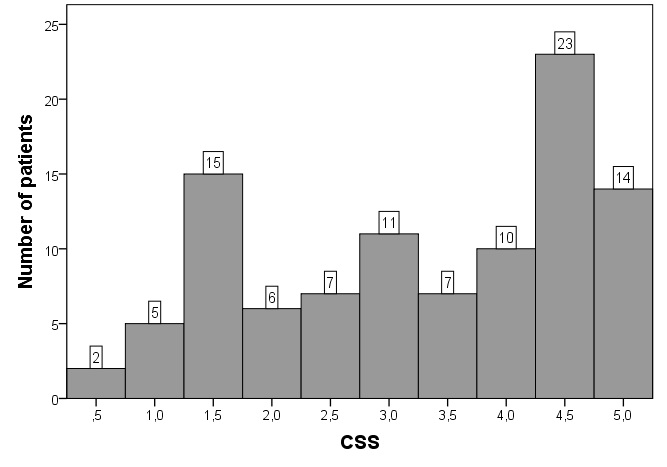


(a)


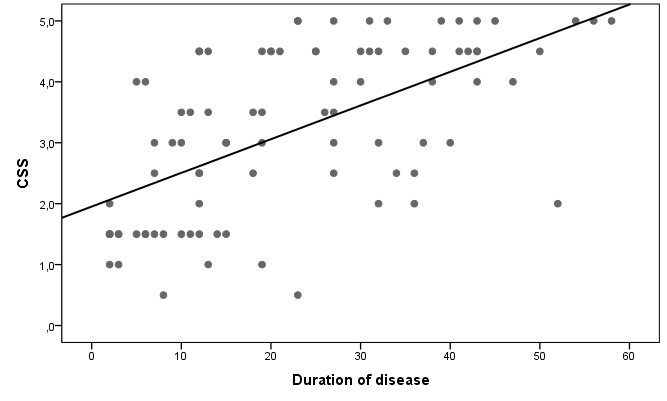


(b)

**Table S2** – Number and proportion of patients according to respiratory pattern (restrictive and non-restrictive), severity of respiratory involvement (FVC < 50% and FVC ≥ 50%) and age of onset (normal onset and early onset). Percentages refer to number of patients in each group of age of onset.

|  | **Respiratory pattern (n = 82)** | | | |
| --- | --- | --- | --- | --- |
|  | **Restrictive**  **(n = 31)** | | | **Non-restrictive**  **(n =52)** |
|  | **< 50%** | $\boldsymbol{\geq}$ **50%** | | |
| **Normal onset**  **(n = 77)** | 10 (13.0%) | 16 (20.8%) | | 51 (66.2%) |
| **Early onset**  **(n = 6)** | 4 (66.7%) | 1 (16.7%) | | 1 (16.7%) |
|  |  |  | |  |
|  |  | |  |  |

**Figure S2** – Plots of forced vital capacity (FVC) values (% predicted) over time for patients with severe respiratory involvement (SR group) (S2.1. to S2.10) and for patients without respiratory involvement (NSR group) with sleep disordered breathing (S2.11 to S2.15). X axis corresponds to year of first and subsequent FVC measurements. Dashed lines represent regression lines for FVC progression of patients in the SR group. Vertical grey lines indicate NIV start and lighter grey lines indicate NIV stop.

S2.2 #74

S2.1 #68


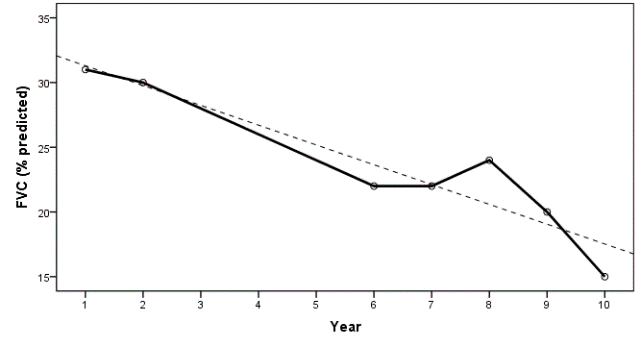

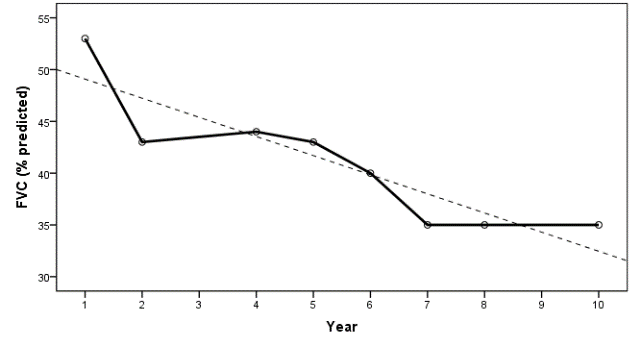


S2.3 #76

S2.4 #84


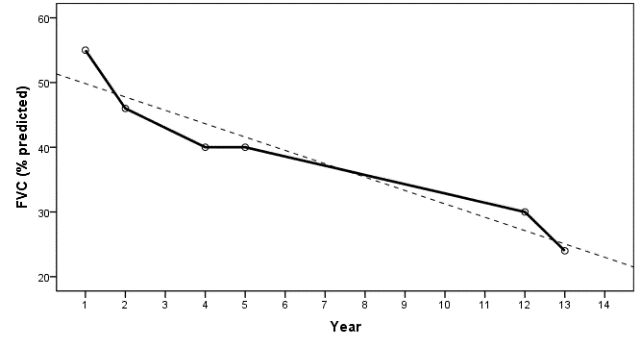

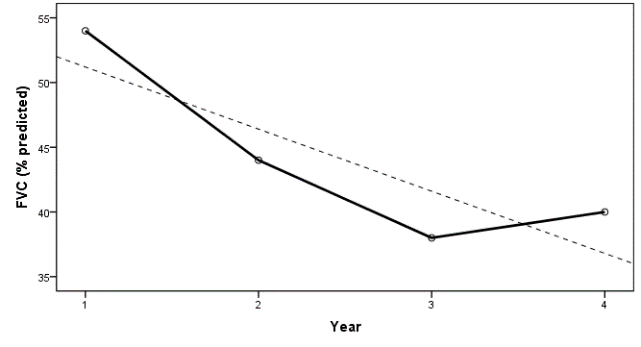


S2.5 #86

S2.6 #87


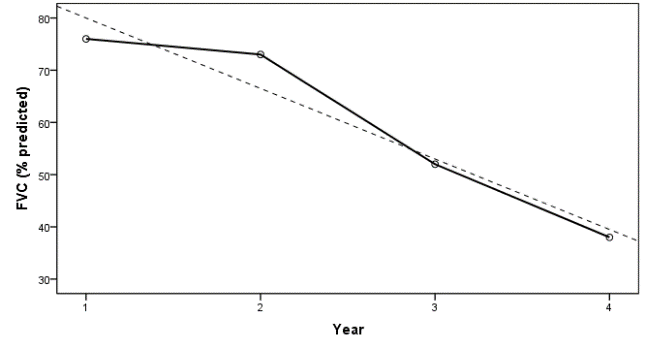


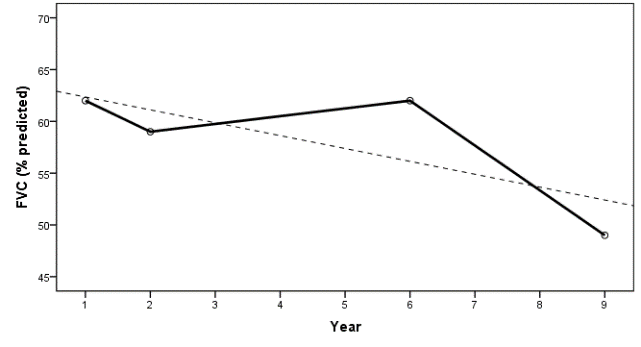


#87

S2.7 #1

S2.8 #27


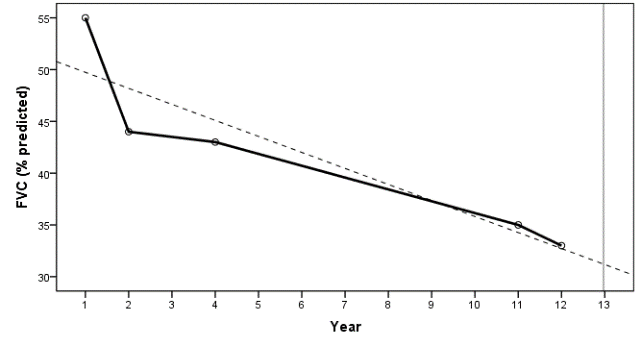

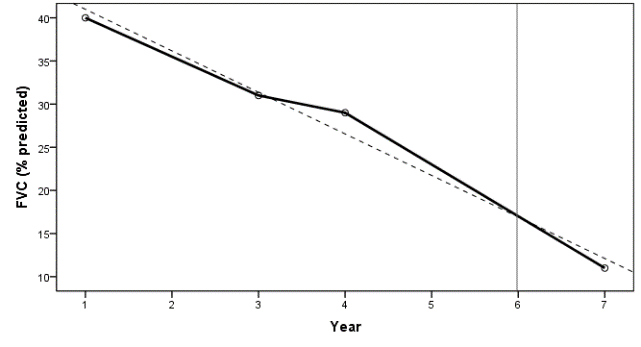


S2.9 #31

S2.10 #100


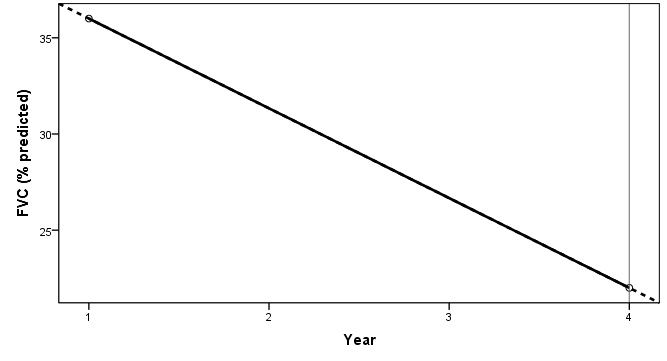

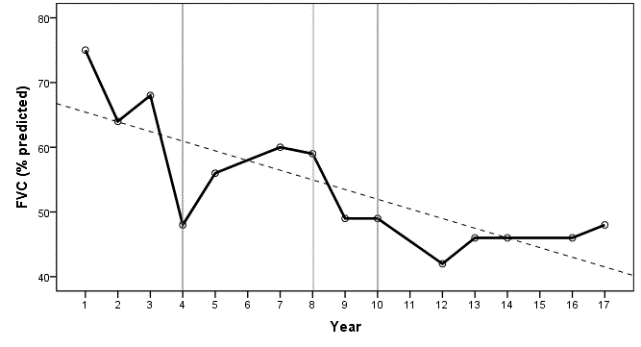


S2.11 #54

S2.12 #61


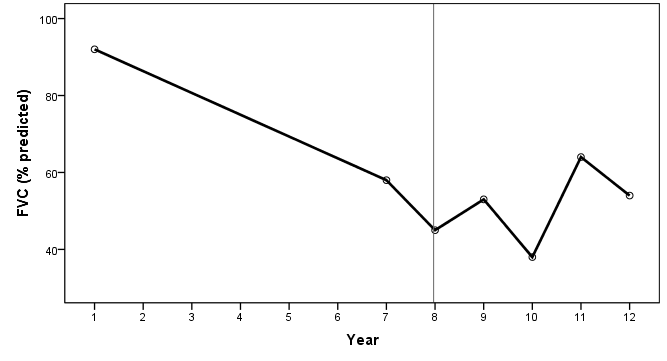

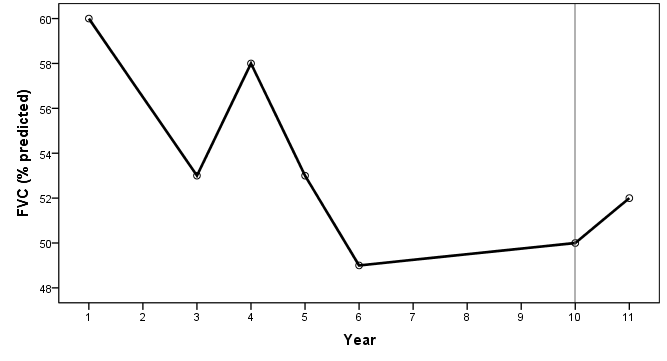


S2.13 #43

S2.14 #47


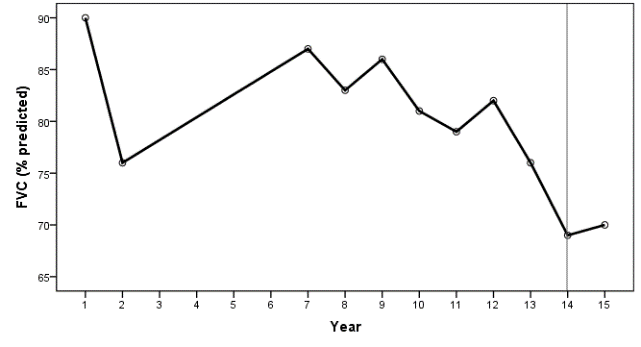

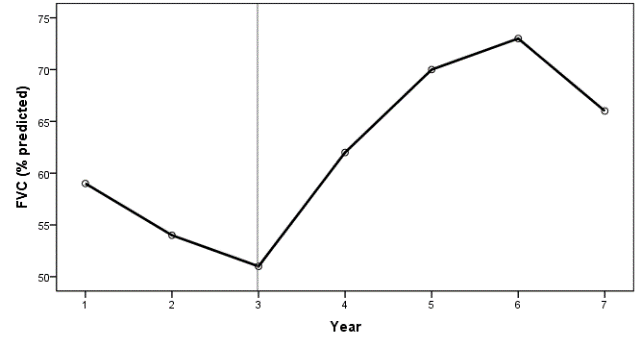


S2.15 #80


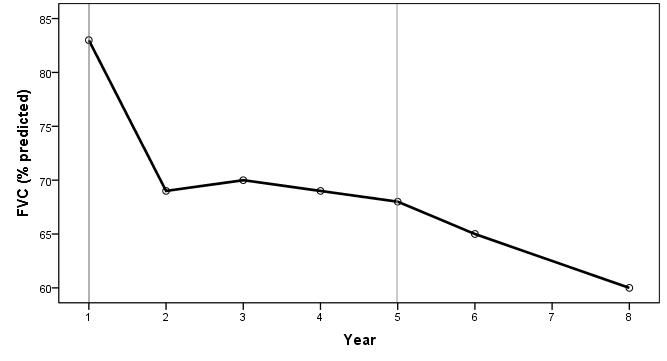

Supplement: Supplementary file 1 — Supplementary material 1 (DOCX 295 kb) [file 415_2017_8525_MOESM1_ESM.docx]
